# Effect of food on the pharmacokinetics and therapeutic efficacy of 4-phenylbutyrate in progressive familial intrahepatic cholestasis

**DOI:** 10.1038/s41598-019-53628-x

**Published:** 2019-11-19

**Authors:** Satoshi Nakano, Shuhei Osaka, Yusuke Sabu, Kei Minowa, Saeko Hirai, Hiroki Kondou, Takeshi Kimura, Yoshihiro Azuma, Satoshi Watanabe, Ayano Inui, Kazuhiko Bessho, Hidefumi Nakamura, Hironori Kusano, Atsuko Nakazawa, Ken Tanikawa, Masayoshi Kage, Toshiaki Shimizu, Hiroyuki Kusuhara, Yoh Zen, Mitsuyoshi Suzuki, Hisamitsu Hayashi

**Affiliations:** 1 Laboratory of Molecular Pharmacokinetics, Graduate School of Pharmaceutical Science, The University of Tokyo, Tokyo, Japan; 2 Department of Pediatrics, Juntendo University Graduate School of Medicine, Tokyo, Japan; 3 Department of Pediatrics, Kindai University Nara Hospital, Nara, Japan; 4 Department of Pediatrics, Osaka University Graduate School of Medicine, Osaka, Japan; 5 Department of Pediatrics, Yamaguchi University Graduate School of Medicine, Yamaguchi, Japan; 6 Department of Pediatrics, Nagasaki University Hospital, Nagasaki, Japan; 7 Department of Pediatric Hepatology and Gastroenterology, Saiseikai Yokohama City Tobu Hospital, Kanagawa, Japan; 8 Clinical Research Center, National Center for Child Health and Development, Tokyo, Japan; 9 Department of Pathology, Kurume University School of Medicine, Fukuoka, Japan; 10 Department of Clinical Research, Saitama Children’s Medical Center, Saitama, Japan; 11 Department of Diagnostic Pathology, Kurume University Hospital, Fukuoka, Japan; 12 Kurume University Research Center for Innovative Cancer Therapy, Fukuoka, Japan; 13 Department of Diagnostic Pathology, Kobe University, Hyogo, Japan

**Keywords:** Clinical pharmacology, Liver diseases, Paediatric research

## Abstract

Progressive familial intrahepatic cholestasis (PFIC), a rare inherited disorder, progresses to liver failure in childhood. We have shown that sodium 4-phenylbutyrate (NaPB), a drug approved for urea cycle disorders (UCDs), has beneficial effects in PFIC. However, there is little evidence to determine an optimal regimen for NaPB therapy. Herein, a multicenter, open-label, single-dose study was performed to investigate the influence of meal timing on the pharmacokinetics of NaPB. NaPB (150 mg/kg) was administered orally 30 min before, just before, and just after breakfast following overnight fasting. Seven pediatric PFIC patients were enrolled and six completed the study. Compared with postprandial administration, an approved regimen for UCDs, preprandial administration significantly increased the peak plasma concentration and area under the plasma concentration-time curve of 4-phenylbutyrate by 2.5-fold (95% confidential interval (CI), 2.0–3.0;P = 0.003) and 2.4-fold (95% CI, 1.7–3.2;P = 0.005). The observational study over 3 years in two PFIC patients showed that preprandial, but not prandial or postprandial, oral treatment with 500 mg/kg/day NaPB improved liver function tests and clinical symptoms and suppressed the fibrosis progression. No adverse events were observed. Preprandial oral administration of NaPB was needed to maximize its potency in PFIC patients.

## Introduction

Progressive familial intrahepatic cholestasis (PFIC) is an autosomal recessive inherited liver disease. Intractable itching, jaundice, and growth retardation due to persistent intrahepatic cholestasis appear during infancy in patients with PFIC. Its exact prevalence is unknown, but it is estimated to be between 1 in 50,000 and 1 in 100,000 births^1,2^. This disease is classified into five types (PFIC1–5) according to gene that causes the disease^1–6^. PFIC1 and 2 are predominant subtypes of PFIC with normal levels of serum gamma glutamyl transferase (normal-GGT PFIC). These subtypes have a genetic deficiency in the *ATP8B1* and *ABCB11*, which encode an aminophospholipid flippase expressed in many tissues and a bile salt export pump (BSEP) that mediates the biliary excretion of bile acids from hepatocytes, respectively^2,3,6^.

Normal-GGT PFIC progresses to liver failure and death before adulthood^7,8^. No effective medical therapy for this disease is currently available^8^. Even liver transplantation is insufficient to overcome normal-GGT PFIC because of ongoing graft steatosis and fibrosis in PFIC1^9^ and recurrent graft failure in some patients with PFIC2^10–12^. We and other groups have published experimental evidence that sodium 4-phenylbutyrate (NaPB), a drug approved for treating urea cycle disorders (UCDs), has another newly identified pharmacological effect: it increases the hepatocanalicular expression of BSEP^13–15^. Furthermore, our group and Gonzales *et al*. showed that treatment with NaPB improved biochemical parameters and liver histology in PFIC2 patients with mutations in *ABCB11* that severely affect the hepatocanalicular expression of BSEP but not its transport activity^14,16–19^. We also found that this drug eliminates intractable itching in patients with PFIC1^20^ in which *ATP8B1* deficiency induces a decrease in both the expression and function of BSEP^21,22^. However, another observational study reported that monotherapy with NaPB had little therapeutic effect in two patients with PFIC2^23^.

The originally described pharmacological action of NaPB involves generating an alternative pathway of nitrogen deposition that replaces the urea cycle through urinary excretion of 4-phenylacetylglutamine (PAG). PAG is formed through the conjugation of glutamine with 4-phenylacetate (PA), a metabolite of 4-phenylbutyrate (PB), leading to a reduction in the plasma ammonia level. Therefore, NaPB has a beneficial effect in UCDs, which are a group of inborn errors of hepatocyte metabolism involved in urea synthesis, resulting in the accumulation of ammonia in the blood at toxic levels. NaPB therapy has been the standard treatment for the long-term management of UCDs for over 20 years. The approved regimen dictates that it is taken with or immediately after a meal^24^. However, this regimen is not supported by specific clinical evidence. Little information about the pharmacokinetics of NaPB, other than its metabolism and excretion pathway, is available^24^. Herein, we performed a multicenter, open-label, single dose study of NaPB in seven patients with normal-GGT PFIC to investigate the influence of meal timing on the pharmacokinetics (PK) of NaPB, and showed that food intake before the administration of NaPB markedly reduced the systemic exposure to PB. We then, over 27 months, assessed the optimal regimen for NaPB in the treatment of normal-GGT PFIC in two patients with PFIC2, through biochemical and liver histological analysis and safety assessments.

## Results

### Meal timing effect on the PK of PB

**Figure 1:**
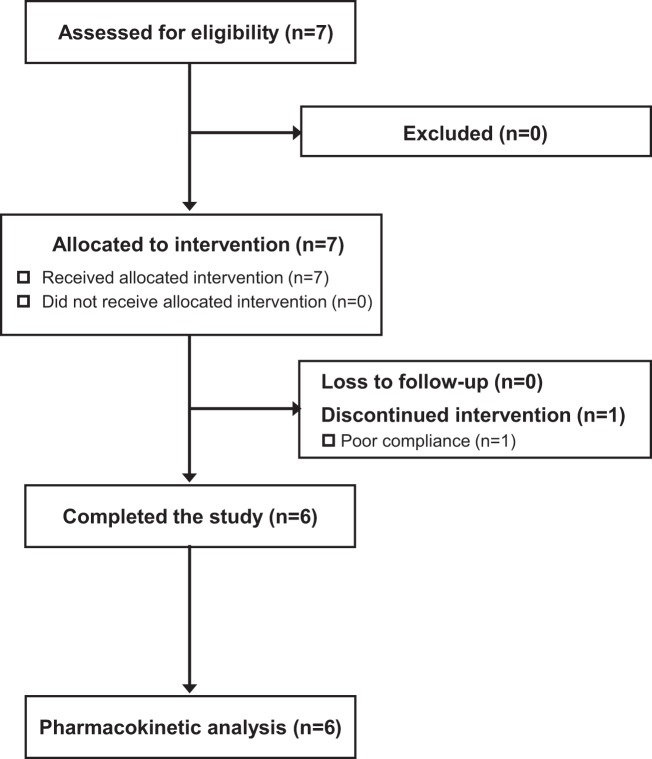
Eligibility and follow-up of patients in the PK study.

Seven patients were diagnosed with normal-GGT PFIC as described in Methods and enrolled in the PK study between November 2016 and March 2017 (Table 1). The subjects comprised three boys and four girls, and the mean ± SD values of their age, height, and body weight were 4.6 ± 2.1 years old (range, 1.5–8.0 years), 89.3 ± 13.4 cm (range, 67.3–109.2 cm), and 13.0 ± 4.0 kg (range, 6.6–19.2 kg). All except Patient 3 were Japanese. No clinically undesirable signs or symptoms attributable to the administration of NaPB were detected during the PK study. All subjects other than Patient 5 completed the study. The PK data concerning NaPB administration just after breakfast were missing in Patient 5 because he refused to take NaPB after breakfast. Therefore, his data were excluded from the PK analysis (Fig. 1).

**Table 1:** Patient demographic characteristics.

Diagnosis		Diagnostic findings		Patient 1	Patient 2	Patient 3	Patient 4	Patient 5	Patient 6	Patient 7
		PFIC1	PFIC2	PFIC2	PFIC2	PFIC2	PFIC1	PFIC1	PFIC1	PFIC-like
**Gene**										
Causal gene		*ATP8B1*	*ABCB11*	*ABCB11*	*ABCB11*	*ABCB11*	*ATP8B1*	*ATP8B1*	*ATP8B1*	ND
Gene mutation	Allele 1			ex.5c.386G>A	ex.5 c.386G>A	ex.26 c.3692G>A	ex.5 c.461_462insT	ex.25 c.3033-34del	ex.28 c.3579_3589del	ND
	Allele 2			ex.5c.386G>A	ex.14 c.1460G>A	ex.26 c.3692G>A	ex.19 c.2124_2125insGAGCTACAGCTATTGAAGGC	ND	ND	ND
**Demographics**										
Age				3y6m	3y10m	5y5m	1y5m	6y1m	8y0m	4y5m
Sex				Girl	Girl	Girl	Girl	Boy	Boy	Girl
Race				Japanese	Japanese	Pakistani	Japanese	Japanese	Japanese	Japanese
Body height (cm)				78.4	96.9	88.5	67.3	91.0	109.2	94.0
Body weight (kg)				9.4	14.8	12.5	6.6	14.3	19.2	14.5
**Characteristics**										
Age of onset		2m^*1^	2m^*1^	2m	1m	2m	4m	2m	3m	5m
Cholestasis		+[100%]	+[100%]	+	+	+	+	+	+	+
Intractable pruritus		+[100%]^*2^	+[100%]^*2^	+	+	+	+	+	+	+
Hepatomegaly		+[100%]^*2^	+[97%]^*2^	+	+	+	+	+	+	+
Jaundice		+[73%]^*1^	+[70%]^*1^	+	+	+	+	+	+	—
Splenomegaly		+[31%]^*2^	+[41%]^*2^	+	+	—	—	+	—	+
Gallstones		+[0%]^*1^	+[32%]^*1^	—	+	—	—	—	—	—
Diarrhea		+[61%]^*1^	+[20%]^*1^	+	—	+	—	+	—	+
Steatorrhea		+[NA]	+[NA]	+	+	+	—	—	—	—
Sensorineural deafness		+[31%]^*1^	+[0%]^*1^	—	—	—	—	—	—	—
Pneumonia		+[13%]^*1^	+[1%]^*1^	—	—	+	—	—	—	—
Pancreatitis		+[12%]^*1^	+[1%]^*1^	—	—	—	—	—	—	—
Bleeding		+[0%]^*2^	+[8%]^*2^	—	+	+	—	—	+	+
Rickets		+[46%]^*1^	+[12%]^*1^	—	—	—	+	+	—	—
Failure to thrive		+[90%]^*1^	+[59%]^*1^	+	—	+	+	+	—	+
Short stature		+[NA]	+[NA]	+	—	+	+	+	+	+
Weight-for-height		> Normal [NA]	> Normal [NA]	> Normal	> Normal	> Normal	< Normal	> Normal	> Normal	> Normal
Mental retardation		+[NA]	+[NA]	+	—	—	—	—	—	—
**Serum assay**										
AST (IU/L)	Peak (age)	<2 × Normal^*1, 3^[NA]	Elevated^*1^[NA]	1,276 (5m)	652 (4m)	895 (5y2m)	163 (5m)	128 (2y1m)	98 (7m)	160 (3y10m)
	Current			56	33	338	93	87	68	50
ALT (IU/L)	Peak (age)	<2 × Normal^*3^[NA]	Elevated^*3^[NA]	1,457 (5m)	890 (4m)	439 (6m)	104 (1y3m)	99 (2y3m)	123 (7m)	208 (3y10m)
	Current			46	19	155	84	44	76	55
GGT (IU/L)	Peak (age)	Low to normal^*3^[NA]	Low to normal^*3^[NA]	118 (1y10m)	33 (5m)	39 (5y1m)	59 (1y1m)	70 (1y1m)	33 (6y5m)	38 (1y11m)
	Current			33	17	30	39	30	28	26
T-Bil (μmol/L)	Peak (age)	Elevated^*3^[NA]	Elevated^*3^[NA]	178.7 (1y11m)	105.8 (1y8m)	196.7 (5y2m)	200.1 (5m)	333.5 (4m)	388.2 (6y5m)	8.6 (2y6m)
	Current			12.5	16.2	65.0	47.9	109.4	71.8	6.8
D-Bil (μmol/L)	Peak (age)	Elevated^*3^[NA]	Elevated^*3^[NA]	80.8 (1y11m)	47.7 (1y8m)	102.4 (5y2m)	102.4 (5m)	180.3 (4m)	194.2 (6y5m)	3.2 (2y6m)
	Current			4.4	4.1	33.1	21.0	43.6	32.7	2.1
TBA (μmol/L)	Peak (age)	Elevated^*1, 3^[NA]	Elevated^*1^[NA]	529.1 (1y2m)	456.2 (1y11m)	357.1 (5y1m)	425 (9m)	351.8 (2y11m)	268.9 (4y8m)	245.4 (4y1m)
	Current			297.6	176.7	NA	297.1	174.4	87.2	145.8
ALP (IU/L)	Peak (age)	Normal^*1^[NA]	Normal (lower limit)^*1^[NA]	1,956 (7m)	2,362 (1y1m)	3,550 (5m)	3,206 (5m)	3,740 (5y6m)	3,943 (8m)	1,575 (1y8m)
	Current			1,525	1,428	NA	2,296	2,018	1,253	1,077
Alb (g/dL)	Nadir (age)	Low to normal^*1^[NA]	Low^*1^[NA]	4.3 (1y3m)	3.1 (1y8m)	1.6 (5y1m)	3.0 (1y1m)	2.5 (1y1m)	2.7 (1y6m)	3.7 (4y5m)
	Current			4.8	4.2	3.0	3.3	3.2	3.7	3.7
TG (mg/dL)	Peak (age)	Elevated^*1^[NA]	Elevated^*1^[NA]	572 (1y6m)	314 (1y3m)	302 (5y1m)	319 (10m)	455 (8m)	358 (6y5m)	434 (4y1m)
	Current			177	71	—	150	152	110	345
T-Cho (mg/dL)	Peak (age)	<2 × Normal^*3^[NA]	<2 × Normal^*3^[NA]	256 (3m)	232 (1y8m)	259 (5y5m)	177 (10m)	235 (2m)	176 (8m)	280 (3y2m)
	Current			159	106	209	129	163	105	230
**Treatment**										
Surgical procedure (age)				—	—	—	—	—	PIBD (1y6m)	—
Liver transplantation (age)				—	—	—	—	—	—	—

Alb, albumin; ALP, alkaline phosphatase; ALT, alanine aminotransferase; AST, aspartate transaminase; D-Bil, direct bilirubin; GGT, gamma-glutamyl transpeptidase; NA, not available; ND, not detected; PIBD, partial internal biliary diversion; PFIC, progressive familial intrahepatic cholestasis; TBA, total bile acid; T-Bil, total bilirubin; T-Cho, total cholesterol; TG, triglyceride.

*1: Pawlikowska L *et al*., J Hepatol. 2010 Jul;53(1):170–8.; *2: Davit-Spraul A *et al*., Hepatology. 2010 May;51(5):1645–55.; *3: Suchy FJ, Sokol RJ, Balistreri WF. Liver Disease in Children, 4th edition. England: Cambridge University Press; 2014: 199–215.

**Figure 2:**
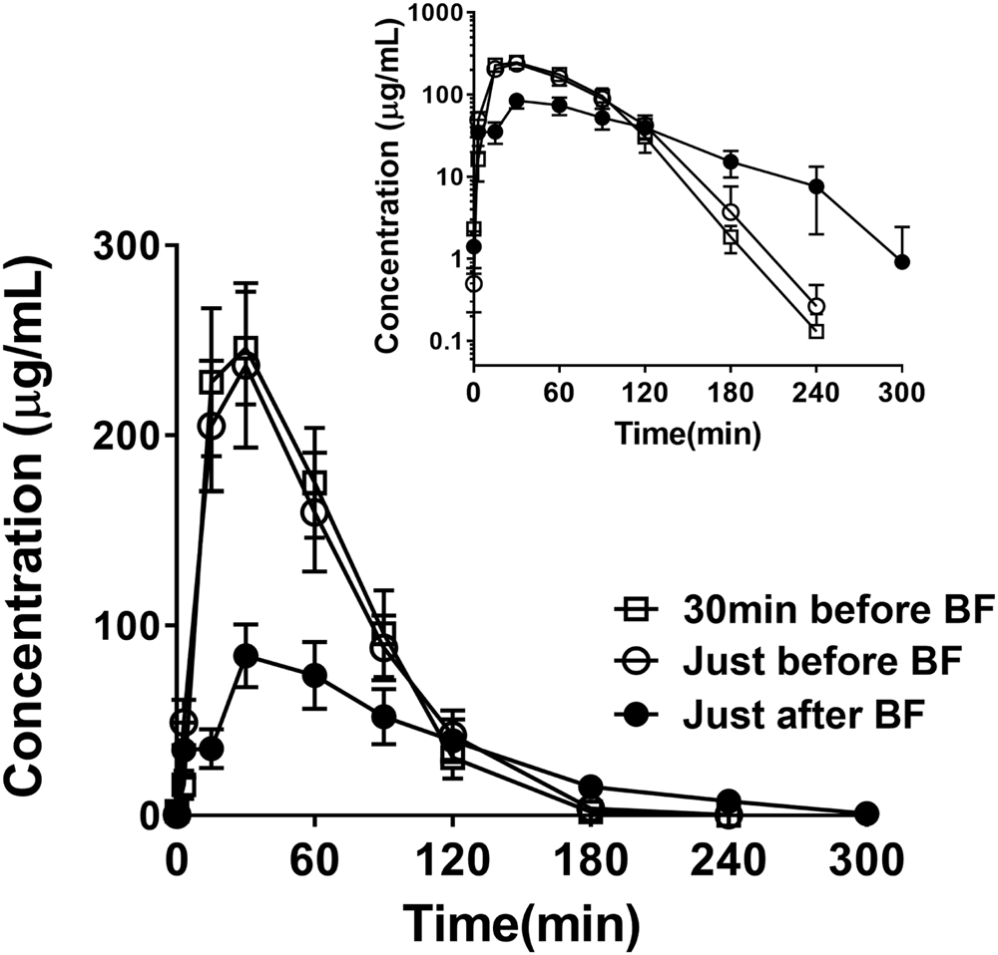
Effect of meal timing on PB systemic exposure after oral administration of NaPB in patients with normal-GGT PFIC. NaPB (150 mg/kg) was administered orally to patients with normal-GGT PFIC 30 min before, just before (<10 min), and just after (<10 min) breakfast following an overnight fast. Each regimen was separated by a washout period of more than 24 h. Plasma concentrations of PB were determined at the times shown. Data concerning all the patients excluding Patient 5, who refused to take NaPB after breakfast, are shown as means ± SEM (n = 6) of the plasma concentrations. The inset depicts the same data on a logarithmic scale. Plasma concentrations of PB at 300 min after the preprandial dosing of NaPB were below the lower limit of quantification. BF, breakfast.

**Figure 3:**
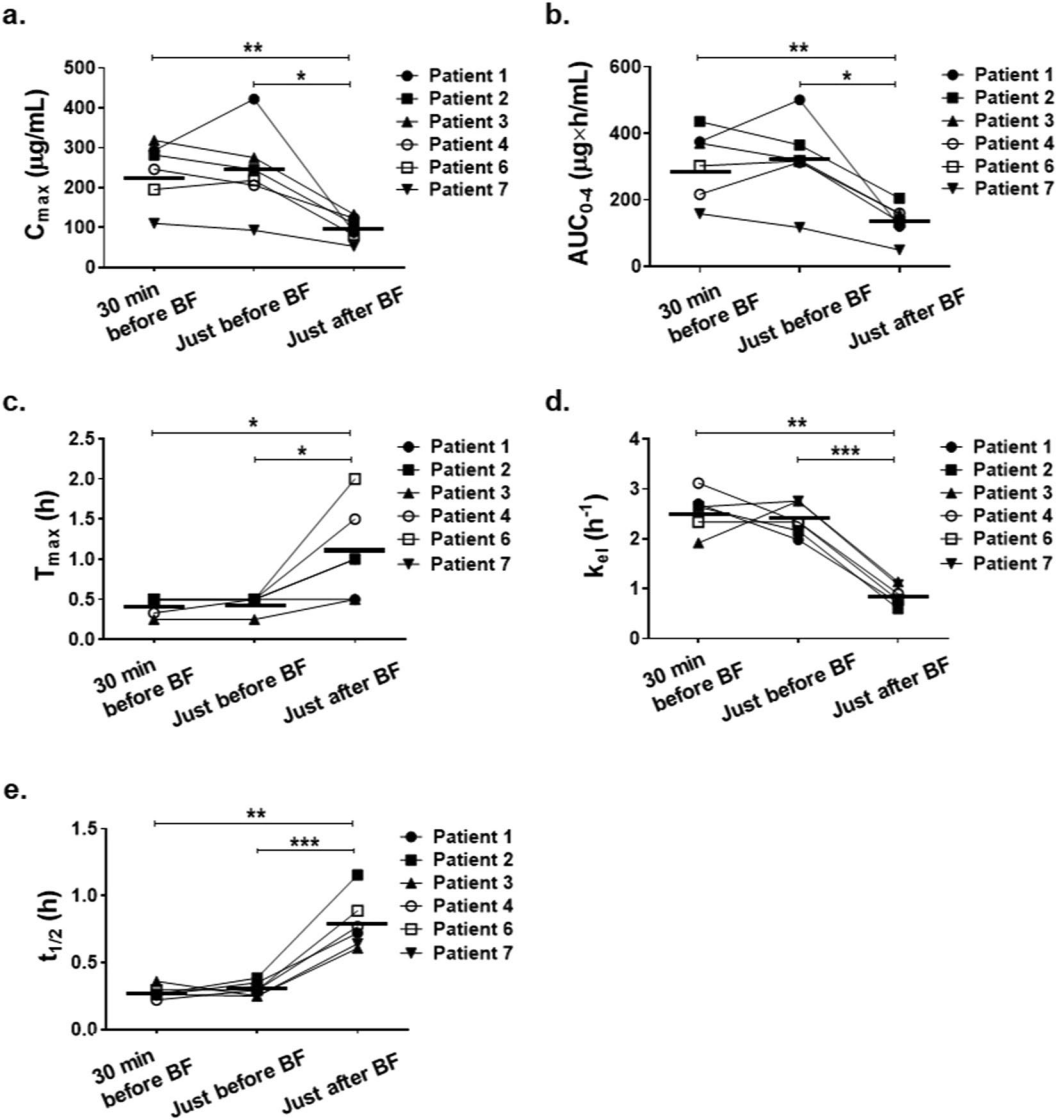
Effect of meal timing on the PK parameters of PB in patients with normal-GGT PFIC. The C_max_ (**a**), AUC_0–4_ (**b**), T_max_ (**c**), k_el_ (**d**), and t_1/2_ (**e**) values of PB were calculated as described in the Methods. The plots represent these parameters for individual patients; the points representing the same patient are connected to one another by lines. The horizontal line in each column indicates the mean values. BF, breakfast. *P < 0.05; **P < 0.01; ***P < 0.001 vs just after BF.

The mean plasma concentration–time curves after a single oral dose of 150 mg/kg NaPB at each time point relative to the meal are shown in Fig. 2. The PK parameters for each patient are shown in Fig. 3. Food intake before the administration of NaPB markedly reduced the plasma concentration of PB and delayed its elimination from blood. There was no significant difference between the administration of NaPB 30 min before or just before breakfast in terms of these parameters. The maximum plasma concentration (C_max_) of PB and the area under the plasma concentration–time curve from time 0 to 4 h after the administration of NaPB (AUC_0–4_) of PB for NaPB administration just after breakfast were 2.47 times (95% confidential interval (CI), 1.96–2.97; P = 0.003) and 2.43 times (95% CI, 1.66–3.21; P = 0.005) lower than those for administration just before breakfast, and 2.54 times (95% CI, 1.31–3.77; P = 0.029) and 2.52 times (95% CI, 1.39–3.64; P = 0.016) lower than those for administration 30 min before breakfast (Table 2). The time to reach C_max_ (T_max_) of PB after postprandial administration occurred later than after preprandial administration. The elimination rate constant (k_el_) of PB was markedly decreased by food intake before administration of NaPB. Consequently, the elimination half-life (t_1/2_) of PB for administration just after breakfast was 3.25 times (95% CI, 2.09–4.41; P = 0.001) and 3.03 times (95% CI, 2.36–3.70; P < 0.001) longer than for administration just before and 30 min before breakfast, respectively.

**Table 2:** PK parameters of NaPB administered orally at 150 mg/kg in patients with normal-GGT PFIC.

Regimen	30 min before BF	Just before BF	Just after BF	30 min before BF to just after BF		Just before BF to just after BF	
				Ratio	P value	Ratio	P value
C_max_ (μg/mL)	240.8 (159.9–321.6)	243.2 (130.7–355.8)	97.8 (66.8–128.9)	2.47 (1.96–2.97)	0.003	2.54 (1.31–3.77)	0.029
AUC_0–4_ (μg×h/mL)	309.7 (199.6–419.7)	324.1 (191.5–456.8)	136.5 (81.1–191.8)	2.43 (1.66–3.21)	0.005	2.52 (1.39–3.64)	0.016
T_max_ (h)	0.500 (0.250–0.500)	0.500 (0.250–0.500)	1.000 (0.500–2.000)	0.500 (0.222–0.500)	0.042	0.500 (0.250–0.500)	0.028
k_el_ (h^−1^)	2.56 (2.14–2.98)	2.46 (2.20–2.72)	0.85 (0.61–1.09)	3.25 (2.09–4.41)	0.001	3.03 (2.36–3.70)	<0.001
t_1/2_ (h)	0.277 (0.227–0.327)	0.295 (0.243–0.346)	0.870 (0.616–1.124)	0.346 (0.199–0.493)	0.005	0.351 (0.289–0.412)	0.002

Data are shown as mean (95% confidence interval); T_max_ data are shown as median (range). BF, breakfast.

The administration of NaPB with breakfast was tested in Patient 1 and resulted in the same plasma concentration and PK parameters for PB as those obtained through administration just after breakfast (Supplementary Fig. S1).

### *In vitro* assessment of the therapeutic potency of NaPB in Patients 1 and 2

Patients 1 and 2 carried homozygous and compound heterozygous mutations, respectively, in *ABCB11* (Table 1). To assess the therapeutic efficacy of NaPB in both patients, the impact of their *ABCB11* mutations on BSEP was explored using HepG2 cells and HEK293T cells ectopically expressing HA-BSEP^WT^, HA-BSEP^C129Y^, and HA-BSEP^R487H^.

**Figure 4:**
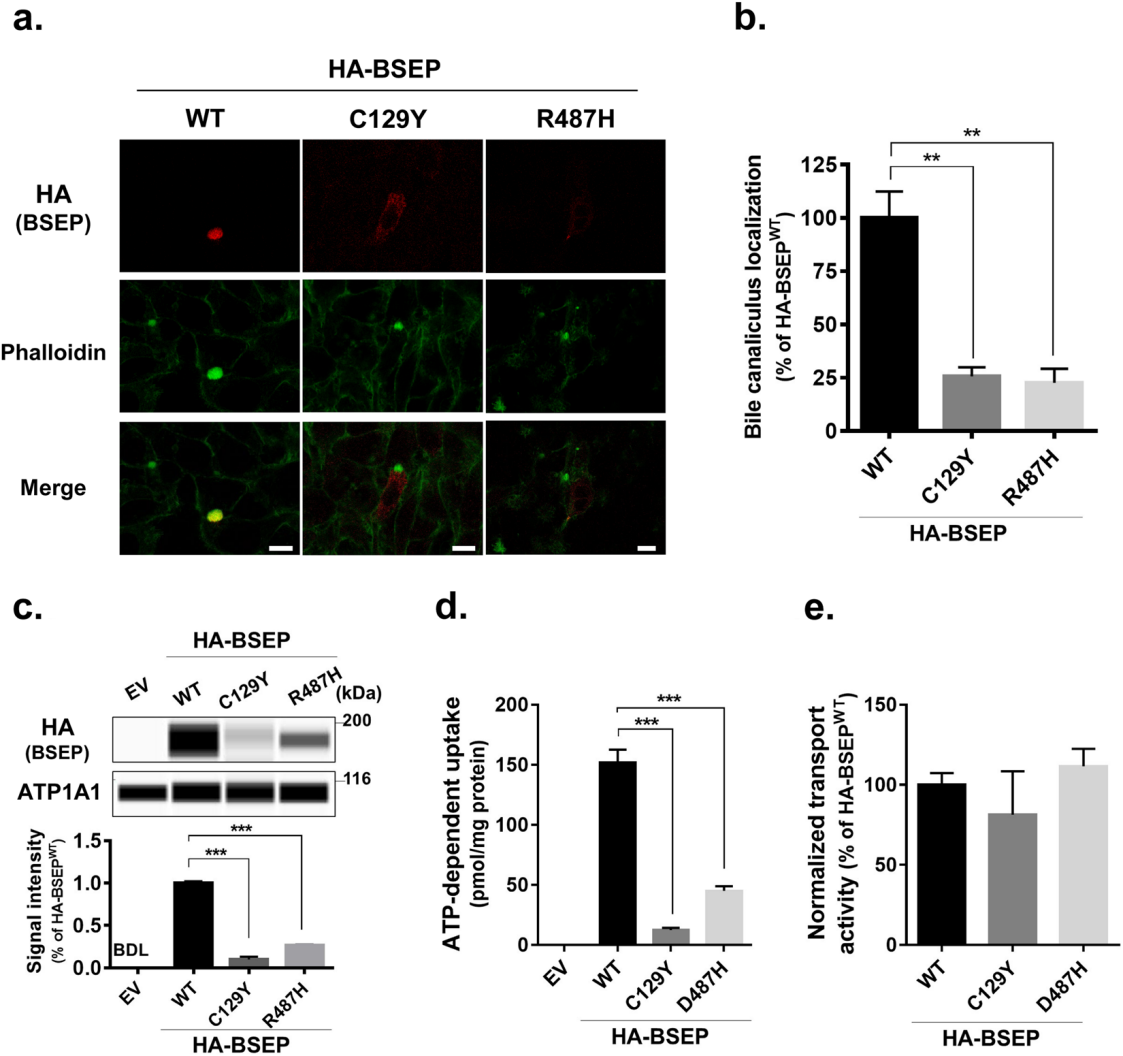
Characterization of c.386 G > A (p.C129Y) and c.1460 G > A (p.R487H) mutations in *ABCB11*. HepG2 cells (**a,b**) and HEK293T cells (**c–e**) were transfected with pShuttle-HA-BSEP^WT^, HA-BSEP^C129Y^, HA-BSEP^R487H^, or a corresponding EV. (**a,b**) Expression and cellular localization of HA-BSEP^C129Y^ and HA-BSEP^R487H^ in HepG2 cells. The cells were immunostained and analyzed through confocal immunofluorescence microscopy. The cellular outline and bile canaliculi were visualized using alexa488-phalloidin. A representative image is shown in (**a**). Yellow in the merged images indicates colocalization. Scale bar: 10 μm. An analysis of the percentage of cells with each form of HA-BSEP at the bile canaliculus is shown in (**b**). A total of 30–40 cells immunostained using an anti-HA antibody were analyzed in each coverslip. (**c–e**) Expression and transport function of HA-BSEP^C129Y^ and HA-BSEP^R487H^ in HEK293T cells. Membrane vesicles prepared from the cells were analyzed through a capillary-based immunoassay (**c**) and subjected to a transport assay with 0.8 μM [^3^H]-TC. ATP-dependent uptake of [^3^H]-TC for 2 min was measured using each batch of membrane vesicles (**d**). Transport activity of [^3^H]-TC by each form of HA-BSEP (**e**) was calculated by normalizing the transport values in (**d**) by the HA-BSEP expression level determined in (**c**). In (**a–e**), a representative result of two independent experiments is shown. Bars represent the mean ± SEM of each experiment in triplicate. **P < 0.01; ***P < 0.001; BDL, below detection limits because of low expression levels; EV, empty vector.

Immunocytochemical analysis using HepG2 cells confirmed that the expression of HA-BSEP^WT^ was predominantly canalicular: it colocalized well with the phalloidin delineating the bile canaliculus. HA-BSEP^C129Y^ and HA-BSEP^R487H^ showed aberrant localization predominantly in endoplasmic reticulum-like structures (Fig. 4a). The number of cells with canalicular expression of either mutant was much lower than that for HA-BSEP^WT^ (Fig. 4b). These results suggest that both mutations induce incomplete folding of BSEP molecules, which are retained in the endoplasmic reticulum and then degraded through proteasomal degradation, as has been reported for other PFIC2-type mutations^18,19^. This leads to a decrease in BSEP expression at the hepatocanalicular membrane.

Consistent with the results obtained in HepG2 cells, expression of HA-BSEP^C129Y^ and HA-BSEP^R487H^ in membrane vesicles from HEK293T cells was 89.9% and 73.4% lower, respectively, than that of HA-BSEP^WT^ (Fig. 4c). The transport assay showed that the ATP-dependent uptake of [^3^H]-taurocholate (TC) per mg of protein was 91.8% and 70.3% lower in HEK293T membrane vesicles expressing HA-BSEP^C129Y^ and HA-BSEP^R487H^ than in those expressing HA-BSEP^WT^ (Fig. 4d). The differences in the expression of each form of HA-BSEP in these membrane vesicles (Fig. 4c) suggest that HA-BSEP^C129Y^ and HA-BSEP^R487H^ maintain their bile-acid transport activity at the same level as HA-BSEP^WT^ (Fig. 4e). This in turn suggests that the increase in hepatic expression of BSEP^C129Y^ and BSEP^R487H^ as a result of NaPB therapy^14,19,25^ is therapeutically efficacious in Patients 1 and 2.

### Biochemical and liver histological changes caused by NaPB therapy in Patients 1 and 2

**Figure 5:**
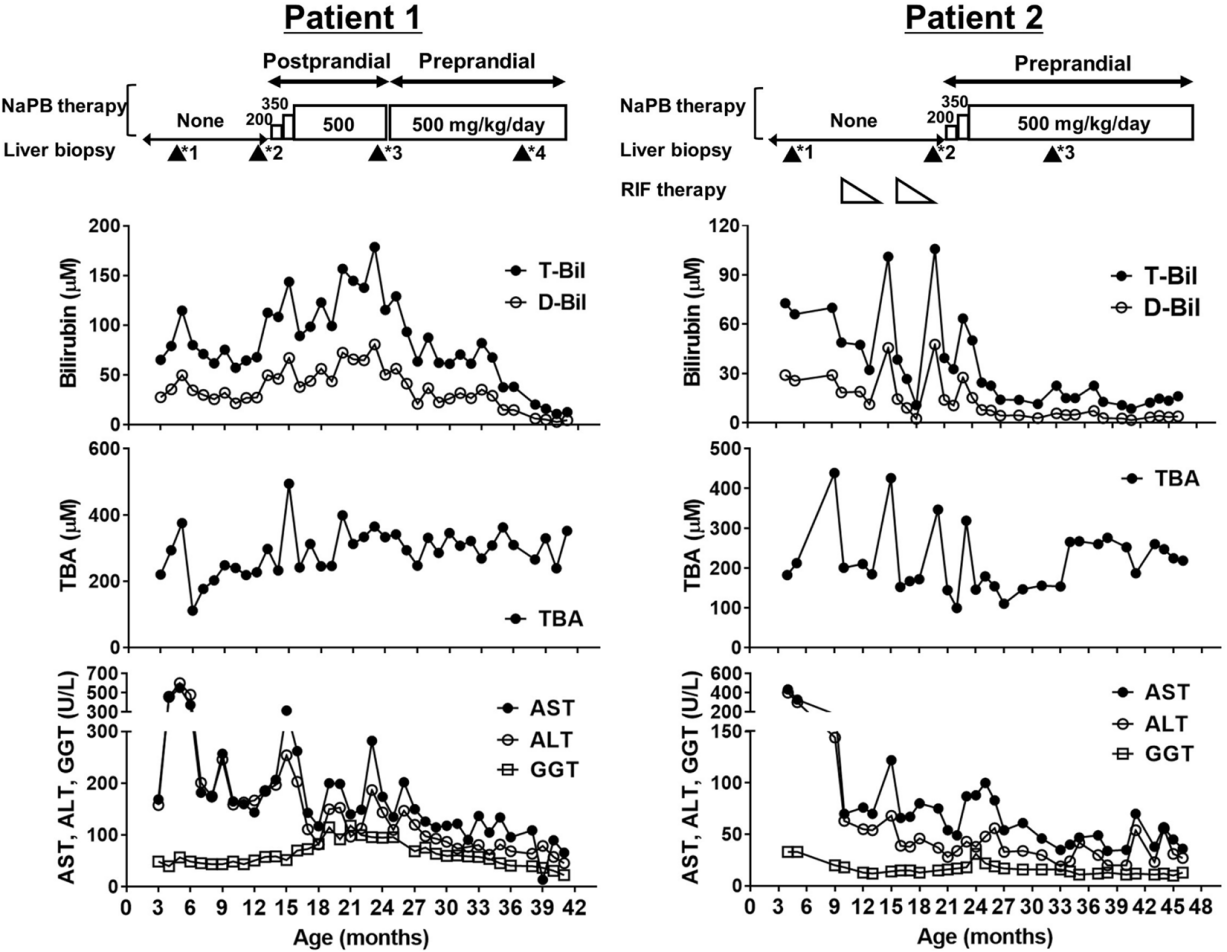
Liver function tests in PFIC2 patients (Patients 1 and 2) before and during the course of NaPB therapy. Patients 1 and 2 underwent NaPB therapy as described in the Methods. Serum T-Bil, D-Bil, TBA, AST, ALT, and GGT levels were monitored. The dosage and regimen of NaPB administration during the course of therapy is shown at the top. Closed triangles represent times when a liver biopsy was performed. The number at the upper right of the triangles corresponds to that of age in Fig. 6. RIF, rifampicin; TBA, total bile acids.

Because *in vitro* assessment indicated the possible therapeutic efficacy of NaPB in Patients 1 and 2 (Fig. 4), these two patients started NaPB therapy at 14 and 22 months of age, respectively. For Patient 1, NaPB was administered orally during or just after meals in accordance with the approved guidelines for UCDs, and for Patient 2 it was administered before meals. The daily dose of NaPB started at 200 mg/kg/day divided into three doses; after 1 month, the dose was increased to 350 mg/kg/day, which was maintained for an additional month. Because neither a sufficient therapeutic effect nor any side effects were observed, the dose was increased to 500 mg/kg/day, the upper limit of the approved dose, and this was maintained for the next 12 and 25 months in Patients 1 and 2, respectively. During this time, the serum levels of total bilirubin (T-Bil) and direct bilirubin (D-Bil), aspartate aminotransaminase (AST), and alanine aminotransaminase (ALT) declined and reached the reference range (T-Bil < 20 μM; D-Bil < 4.3 μM; AST < 55 U/L; ALT < 40 U/L) in Patient 2, but not in Patient 1 (Fig. 5). Because of this, and based on the results of the PK study (Figs. 2, 3 and Table 2), Patient 1 was switched to preprandial oral administration of 500 mg/kg/day NaPB to increase PB systemic exposure and enhance PB therapeutic efficacy. The patient’s serum T-Bil and D-Bil levels gradually declined to within the reference range by 10 months after the change in regimen (Fig. 5). Serum AST and ALT levels also decreased and reached 66 and 46 U/L, respectively, close to the reference ranges (Fig. 5).

In both patients, the improvement in liver function test results was accompanied by the disappearance of their jaundice and intractable itching, which eliminated their sleep disturbance. In contrast, the concentration of serum total bile acid (TBA) remained unchanged during the period of NaPB therapy, probably because of the co-administration of ursodeoxycholic acids. No severe side effects were observed during NaPB therapy.

**Figure 6:**
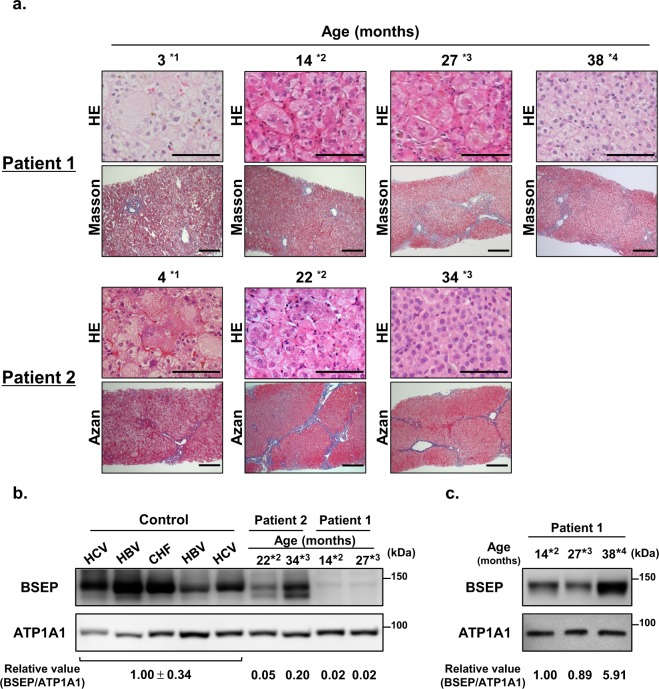
Liver histology and hepatic BSEP expression in PFIC2 patients (Patients 1 and 2) before and during the course of NaPB therapy. (**a**) Liver histology. The liver sections were subjected to HE, Azan, or Masson’s trichrome staining. A typical image under each condition is shown. Original magnification; 400× (HE staining) and 100× (Azan and Masson’s trichrome staining). Bar, 100 μm. (**b,c**) Hepatic BSEP expression. The prepared membrane fractions (5 μg) were analyzed with those from age-matched control subjects through immunoblotting. The signal intensity of BSEP relative to that of ATP1A1 is presented below the panel. CHF, congenital hepatic fibrosis; HBV, hepatitis B virus; HCV, hepatitis C virus.

Liver biopsies were performed at 3 months or 4 months of age for diagnosis and at 14, 27, and 38 months or 22 and 34 months of age to evaluate disease progression in Patients 1 and 2, respectively. In both patients, histological analysis at diagnosis showed cholestasis, giant cell transformation of hepatocytes, and fibrosis, liver histology compatible with PFIC2. Aggravation of these features with age was confirmed until onset of preprandial oral administration of NaPB. Preprandial oral administration of NaPB for 1 year markedly relieved cholestasis and hepatocyte swelling and suppressed the progression of fibrosis (Fig. 6a). Before commencement of preprandial oral administration of NaPB at 27 and 22 months of age in Patients 1 and 2, respectively, BSEP expression in the liver membrane fractions was <5% of that in age-matched control subjects. Preprandial oral NaPB therapy partly restored BSEP expression, although it remained at 12–20% of that in age-matched control subjects (Fig. 6b,c).

## Discussion

In the present study, we showed that preprandial oral administration, rather than the prandial or postprandial administration approved for treating UCDs, was needed for NaPB therapy in normal-GGT PFIC, because food intake before the administration of NaPB markedly reduced systemic exposure to PB (Fig. 2) and eliminated its therapeutic potency (Figs. 5 and 6). In addition, no severe adverse events were observed during preprandial oral NaPB therapy.

After intestinal absorption, PB is taken up into hepatocytes and predominantly converted to PA through β-oxidation. PA forms PAG through conjugation with glutamine and is then excreted into the urine^26^. A PK study of a single oral administration of NaPB (150 mg/kg) in seven patients with normal-GGT PFIC demonstrated that postprandial administration markedly decreased the C_max_ and AUC_0–4_ of PB by approximately 60% compared with preprandial administration (Figs. 2, 3 and Table 2). The longer time until T_max_ observed with postprandial oral administration (Table 2) suggests that food intake affects absorption of PB by enterocytes as well as the ionization of PB by changes of the gastric pH and/or protein binding, leading to reduced intestinal absorption and systemic exposure of PB. The effect of food was also observed in the elimination of PB from the blood with its t_1/2_ value more than 2.5 times greater with postprandial administration than with preprandial administration (Figs. 2, 3 and Table 2). Considering the elimination process of PB, this could stem from the influence of food ingestion on the hepatic uptake of PB, rather than PB β-oxidation to form PA in hepatocytes, because both PA and PB increase BSEP expression in the liver^27^. In that case, hepatic exposure to PB after postprandial oral administration of NaPB could be less than that predicted by its systemic exposure. Alternatively, the delayed elimination phase of PB could be explained by flip-flop pharmacokinetics due to the short half-life of PB.

The results of the current study in the two PFIC2 patients indicated that preprandial oral administration of NaPB, but not prandial or postprandial oral administration, normalized or nearly normalized the results of liver function tests, relieved cholestasis, suppressed the progression of hepatic fibrosis, and markedly improved jaundice, intractable itching, and sleep disturbance (Fig. 5). It is likely that the lack of effective therapeutic outcomes after prandial or postprandial administration of NaPB resulted from reduced hepatic exposure to PB because of the inhibitory effect of food intake on the intestinal absorption of PB, resulting in hepatic concentration that were insufficient to increase the hepatocanalicular expression of BSEP (Fig. 6b,c). This is supported by our previous *in vitro* study showing that the minimum concentration of PB required to increase ectopic BSEP expression in MDCKII cells was 1000 μM (164.2 μg/mL)^14^, which is above the plasma concentration of PB achieved through NaPB administration just after breakfast (Fig. 2).

In our previous study examining the short-term efficacy and safety of NaPB, biological and histological improvements were obtained in another PFIC2 patient (Patient 3) at a dosage of 500 mg/kg/day^19^. This patient’s mother divided the daily dosage of NaPB into four and gave the patient three doses preprandially and between meals, because the patient showed poor compliance with medication given orally in the prandial and postprandial period because of a sense of fullness. In contrast, we tested the short-term effect of NaPB in another two PFIC1 patients including Patient 5, using a regimen of postprandial oral administration, as approved for UCDs^20^. In these patients, NaPB therapy relieved intractable itching and resolved sleep disturbance, but did not improve liver function tests and liver histology. There are four other studies that have examined the efficacy and safety of NaPB in patients with normal-GGT PFIC^16,17,23,28^. Gonzales *et al*. reported that NaPB therapy improved the clinical and biological parameters of cholestasis in three of four PFIC2 patients and helped them to manage their medical condition^16,17^. However, based on observations in two PFIC2 patients, Malatack *et al*. suggested a combined drug regimen including NaPB for PFIC2, because monotherapy with NaPB appeared to be insufficient to improve and maintain the clinical condition of the patients^23^. There is no description in any of these articles concerning how or when NaPB was administered to these patients. Therefore, the inconsistencies between the results of these studies may be explained by the food interaction effects found in the current study. Additional studies with more patients are required to fully understand the therapeutic potency of NaPB in normal-GGT PFIC.

After confirming the optimal protocol to maximize the efficacy and safety of NaPB, it may be desirable to evaluate a combination therapy of NaPB with Maralixibat as previously proposed^23^. Maralixibat blocks the enterohepatic circulation of bile acids by inhibiting the apical sodium-dependent bile acid transporter that is expressed on intestinal epithelial cells and mediates the absorption of bile acids from the intestinal lumen. Therefore, therapy combining Maralixibat with NaPB, which enhances the biliary excretion of bile acids by increasing the hepatocanalicular expression of BSEP, could generate a synergistic therapeutic effect by lowering hepatic bile-acid levels in patients with normal-GGT PFIC, with the exception of those with a loss-of-function mutation in *ABCB11*. Such a combination therapy may become the preferred choice for the treatment of normal-GGT PFIC, replacing the need for partial external biliary diversion and liver transplantation.

A questionnaire survey of 52 patients of UCDs and their caregivers and physicians showed that patients have difficulty taking NaPB because of its odor, taste, and large dosage, which results in poor medication compliance and hence worse therapeutic outcomes^29^. The physicians indicated that the patients with UCDs were prescribed less than the target dosage of NaPB because of concerns about tolerance, administration, and cost. The findings of the current study suggest that in UCDs, preprandial instead of the approved prandial or postprandial administration may make it possible to reduce the clinically effective dosage of NaPB. It is not possible to compare the PK parameters in the current study with those obtained in healthy adults and individuals with other diseases because the relationship between dosing and meal timing was not described in previous studies^26,30^. However, considering that the effects of meal timing on the PK of NaPB were similar in all the participants in the present study (Fig. 3), who varied in age and disease severity (Table 1), it is conceivable that the findings of this study are applicable to UCDs as well as to normal-GGT PFIC.

In conclusion, the present study showed that food intake before the administration of NaPB markedly reduced PB systemic exposure and could eliminate its therapeutic efficacy in patients with normal-GGT PFIC. Therefore, preprandial oral administration of NaPB could be needed to maximize its potency in these patients and decrease the clinically effective dose in patients with UCDs. To determine an optimal NaPB regimen for each disease, preprandial treatment with NaPB should be assessed in future research with careful monitoring of adverse events, such as PA-induced hepatotoxicity^28^.

## Methods

This study was approved by the institutional review boards at the University of Tokyo, Juntendo University, Saiseikai Yokohama City Tobu Hospital, Yamaguchi University Hospital, Osaka University Hospital, and Nagasaki University Hospital, and was performed in accordance with the amended Declaration of Helsinki. Written informed consent was obtained from the parents of each patient prior to enrollment in the study.

### Patients

Seven patients with normal-GGT PFIC participated in the PK study (Table 1). The clinical diagnosis of normal-GGT PFIC was based on the presence of unremitting hepatocellular cholestasis with intractable pruritus, poor growth, and jaundice with normal serum GGT levels, in the first year of life, and the exclusion of type A, B, C, and E hepatitis, biliary atresia, Alagille syndrome, neonatal intrahepatic cholestasis caused by citrin deficiency, Dubin–Johnson syndrome, and Wilson disease. The patients did not suffer from any disease other than cholestasis, as determined through a detailed medical history, full physical examination, vital signs, urinalysis, and blood tests with values within the reference range or deemed to be normal by the clinical investigator.

Sanger sequencing of *ABCB11* and *ATP8B1* and liver biopsies were performed to determine the subtype of PFIC^19,20^. Patients who carried disease-causing mutations in both alleles of *ABCB11* or *ATP8B1* were diagnosed with PFIC2 (Patients 1–3) or PFIC1 (Patient 4), respectively. Patients 5 and 6, in whom only one mutant allele of *ATP8B1* was detected, were diagnosed with PFIC1 because phenotypic analysis using peripheral blood monocyte-derived macrophages indicated an ATP8B1 deficiency^2^. Patient 7 had no mutations detectable through Sanger sequencing of either gene, and therefore was diagnosed with PFIC-like disease. The patients had not undergone any surgical procedures, except Patient 6 who underwent partial internal biliary diversion.

### PK study design

To examine the effect of meal timing on the PK of NaPB in patients with normal-GGT PFIC, a multicenter, open-label, single-dose PK study was conducted in Juntendo University Hospital, Saiseikai Yokohama City Tobu Hospital, and Yamaguchi University Hospital. Eligible participants were children with a confirmed diagnosis of normal-GGT PFIC who had not undergone liver transplantation. Seven relevant patients as described above were identified in a Japanese nationwide survey conducted in 2015. They were enrolled by the physicians in the PK study between 29/11/2016 and 1/3/2018. This study was registered in the UMIN Clinical Trials Registry at http://www.umin.ac.jp/ctr/index.htm (registration ID: UMIN000025037) on the 29/11/2016.

The patients were admitted a day before the PK study and were orally administered with 150 mg/kg NaPB (Buphenyl; OrphanPacific, Tokyo, Japan) 30 min before breakfast, just before (<10 min) breakfast, and just after (<10 min) breakfast following overnight fasting. Each regimen was separated by a washout period of more than 24 h, based on a previous study reporting that the majority of PB and its metabolites disappear from systemic circulation and are excreted into the urine within 24 h after oral administration of NaPB^24,26^. Standard hospital food suitable for the children was served as breakfast, and at 3 and 9 h post-treatment. All patients were prohibited from ingesting any food for at least 3 h after NaPB administration. Treatment with other drugs was maintained during patient participation in the PK study.

Blood samples were collected through a catheter placed in a forearm vein into an EDTA-2Na^+^-pretreated tube prior to, soon after, and 15, 30, 60, 90, 120, 180, 240, and 300 min after drug administration. Blood samples were placed at 4 °C immediately after collection and centrifuged for 15 min at 3,000 rpm to prepare the plasma. The prepared specimens were analyzed to measure PB concentration as described in the supplementary information.

### PK analysis

The C_max_ and T_max_ of PB were determined directly from the observed data. The AUC_0–4_ of PB was calculated using the linear trapezoidal rule. The k_el_ of PB was estimated using a least-squares regression analysis from the terminal post-distribution phase of the concentration–time curve. The t_1/2_ of PB was calculated as 0.693 divided by the k_el_.

### Treatment of the PFIC2 patients with NaPB

The effect of food on the therapeutic efficacy of NaPB was investigated in two patients (Patients 1 and 2) because the other patients participated in another clinical trial after the PK study. Patients 1 and 2 developed hepatocellular cholestasis and jaundice with normal GGT at the age of 1 and 2 months, respectively, and were diagnosed with PFIC2 at 5 and 4 months, respectively, based on genetic analysis and the lack of a detectable signal for BSEP at the canalicular membrane on performing a liver histological analysis. Patient 1 carried a homozygous mutation, c.386 G > A (p.C129Y) in *ABCB11*, the most frequent mutation in Japanese PFIC2 patients^25,31^. Patient 2 carried a compound heterozygous mutation, c.386 G > A (p.C129Y) and c.1460 G > A (p.R487H) in *ABCB11* (see Table 1). Neither patient showed adequate improvement in liver biochemical tests and clinical symptoms with conventional medical treatment such as ursodeoxycholic acid. Patient 2 received two cycles of rifampicin treatment at 9–12 months of age and at 15–18 months of age, with gradually decreasing dosages (10, 7.5, 5, and 1.4 mg/kg/day). Although the medication gradually decreased patient jaundice and serum T-Bil and D-Bil levels and partially alleviated pruritus, the symptoms recurred after rifampicin withdrawal on both occasions (Fig. 5). Liver histological analysis at 22 months of age showed disease progression, compared with that at 4 months of age (Fig. 6a). Based on these observations and the fact that rifampicin is not suitable for long-term treatment because of its potential hepatotoxic effects^32–34^, the patients started NaPB therapy at 14 and 22 months of age, respectively. Before the intervention, the potential therapeutic potency of NaPB was confirmed in both patients through an *in vitro* assessment (Fig. 4) and our previous study^25^. In both patients, treatment with ursodeoxycholic acids and fat-soluble vitamins was maintained during the course of NaPB therapy.

Liver function tests were performed monthly using standard methods. A liver needle biopsy was performed before and during the course of NaPB treatment. Immediately after the sample collection, a part of the liver sample was fixed in 4% formaldehyde at room temperature for histological analysis, and the remaining portion was snap frozen in liquid nitrogen and then stored at −80 °C for the preparation of membrane fractions. A detailed description for the histological analysis and preparation of membrane fractions is presented in the supplementary information.

### Statistical analysis

Data are shown as means ± standard error of the mean (SEM), unless otherwise indicated. Statistical analyses were performed using Prism software (v. 6; GraphPad Software, La Jolla, CA). The data of C_max_, AUC_0–4,_ k_el_, and t_1/2_ values were analyzed through repeated one-way analysis of variance with a post-hoc Dunnett’s test. The T_max_ values were compared using Friedman test with a post-hoc Dunn’s tests. The nonclinical studies were analyzed through one-way analysis of variance with a post-hoc Dunnett’s test. Differences were considered significant at P < 0.05.

## Supplementary information

Supplementary information
