# Supplementary material for: Effect of food on the pharmacokinetics and therapeutic efficacy of 4-phenylbutyrate in progressive familial intrahepatic cholestasis
Source: Sci Rep. 2019 Nov 19;9:17075. doi: 10.1038/s41598-019-53628-x (PMC6863819; doi:10.1038/s41598-019-53628-x)
Supplement: Supplementary file 1 — Supplementary information [file 41598_2019_53628_MOESM1_ESM.pdf]

Supplementary information

Effect of food on the pharmacokinetics and therapeutic efficacy of 4-phenylbutyrate in progressive familial intrahepatic cholestasis

Satoshi Nakano[1, 2, +], Shuhei Osaka[1, +], Yusuke Sabu[1], Kei Minowa[2], Saeko Hirai[2], Hiroki Kondou[3], Takeshi Kimura[4], Yoshihiro Azuma[5], Satoshi Watanabe[6], Ayano Inui[7], Kazuhiko Bessho[4], Hidefumi Nakamura[8], Hironori Kusano[9], Atsuko Nakazawa[10], Ken Tanikawa[11], Masayoshi Kage[12], Toshiaki Shimizu[2], Hiroyuki Kusuhara[1], Yoh Zen[13], Mitsuyoshi Suzuki[2], Hisamitsu Hayashi[1, *]

Methods

Clincal studies

Quantification of PB concentrations in human plasma

20 µL of human plasma was transferred to a 96 deep-well plate and mixed with 80 µL acetonitrile containing the internal standard (1 µg/mL 4-phenylbutyric acid-d11, Toronto Research Chemicals Inc., Ontario, Canada). After vortexing, the mixtures were filtered using a FastRemover for Protein (GL Sciences, Tokyo, Japan). The eluents were diluted 10 times with 0.01% formic acid and subjected to quantitation using an AB SCIEX QTRAP 5500 mass spectrometer (Applied Biosystems, Foster City, CA) equipped with a Prominence high-performance liquid chromatography system (Shimadzu Corporation, Kyoto, Japan), operated in negative electrospray ionization mode. Chromatographic separation was achieved at 40°C using a Gemini C-18 column (150 × 2 mm, 3 µm; Phenomenex,

Torrance, CA) in binary gradient mode at a flow rate of 0.4 mL/min. The mobile phase consisted of acetonitrile with 0.01% formic acid. The acetonitrile concentration was initially 10%; it was then linearly increased to 80% over 1 min and maintained for a further 1.1 min. Finally, the column was re-equilibrated using acetonitrile at a concentration of 10% for 1.8 min. The mass transition was from m/z 162.9 to 90.9 for PB and from m/z 174.1 to 98.0 for the internal standard. The analytical method was validated in terms of selectivity and linearity (0.5–500 μg/mL in human plasma, correlation coefficient (r) ≥0.99).

Histological analysis of human liver specimens

Liver specimens were fixed in 10% formalin and embedded in paraffin; then 4μm sections were prepared using a microtome REM-710 (Yamato Kohki, Saitama Japan), adhered to glass coverslips, and subjected to hematoxylin–eosin (HE), Azan, or Masson's trichrome staining followed by microscopic analysis using an Olympus CX41 (Olympus, Tokyo, Japan) to evaluate the degree of cholestasis, giant cell transformation, inflammation, and fibrosis. Slides were reviewed by three independent pathologists (A.N., K.T., and Y.Z.) with no knowledge of the associated clinical information.

Measurement of protein expression in human liver specimens

Liver specimens from the patients were homogenized in hypotonic buffer (1 mM EDTA, 5 mM sodium phosphate, pH 7.0) supplemented with protease inhibitor cocktails (Merck, Darmstadt, Germany) using a QIAshredder (Qiagen, Valencia, CA), and then centrifuged at 800×g for 10 min at 4°C. The supernatant was ultracentrifuged at 100,000×g for 1 h at 4°C. The resultant pellets were lysed, loaded into wells of an 8% SDS-

polyacrylamide gel with a 3.75% stacking gel, electrophoresed, and subjected to immunoblotting as described previously [1,2]. The intensity of the band indicating each protein was quantified using MultiGauge software (v. 2.0; Fujifilm, Tokyo, Japan).

Nonclincal studies

Plasmids

The pShuttle vector (Clontech, Palo Alto, CA) containing human BSEP cDNA N-terminally tagged with a hemagglutinin antigen (HA) (HA–BSEP$^{wild\ type(WT)}$) was constructed [3] and the c.386G>A (p.C129Y) or c.1460G>A (p.R487H) mutation (HA-BSEP^{C129Y} and HA-BSEP^{R487H}) were introduced by site-directed mutagenesis as described previously [4].

Cell culture and transfection

HEK293T cells were purchased from the American Type Culture Collection (Manassas, VA) (CRL-11268) and HepG2 cells from RIKEN Cell Bank (Tsukuba, Japan) (RCB1886). HEK293T cells were maintained in Dulbecco's modified Eagle's medium (DMEM) (Invitrogen, Carlsbad, CA) supplemented with 10% fetal bovine serum (FBS; Invitrogen) and HepG2 cells in DMEM supplemented with 10% FBS and 1% nonessential amino acids (Invitrogen). Both types of cells were cultured at 37 °C in 5% CO_2 and 95% humidity.

HEK293T cells and HepG2 cells were transfected with the pShuttle vector containing HA-BSEP^{WT}, HA-BSEP^{C129Y}, HA-BSEP^{R487H}, or empty vector (EV) using PEI MAX (Polysciences, Warrington, PA) according to the

manufacturer's instructions and subjected to *in vitro* experiments 48 h after transfection.

Immunocytochemistry

The transfected HepG2 cells were cultured on glass coverslips (Matsunami Glass Ind Ltd, Osaka, Japan), fixed in 4% paraformaldehyde/phosphate buffered saline (PBS) for 10 min, permeabilized in 0.1% saponin/PBS for 10 min, blocked with 3% bovine serum albumin (BSA)/PBS for 30 min, and immunostained together with Alexa 488-conjugated phalloidin (1:100; Cell Signaling Technology, Beverly, MA) and DRAQ5 (1:250; Thermo Fisher Scientific, Waltham, MA) at room temperature. The following antibodies were used: rat anti-HA (1:200; 3F10, Roche, Mannheim, Germany) and Alexa 546-conjugated secondary antibodies (1:200; Invitrogen). The cells were mounted on glass slides with Vectashield mounting medium (Vector Laboratories Inc., Burlingame, CA) and then visualized using a Zeiss LSM 880 confocal laser scanning microscope (Carl Zeiss, Jena, Germany).

Preparation of cell lysates

Cell lysates were prepared from the transfected HEK293T cells using Cell Lysis Buffer (Cell Signaling Technology) according to the manufacturer's instructions. The prepared specimens were analyzed by capillary-based immunoassay.

Preparation of and transport assays with membrane vesicles

Membrane vesicles were prepared from the transfected HEK293T cells as described previously [4,5]. The

isolated membrane vesicles were subjected to transport assays and capillary-based immunoassay. Transport assays were performed using the rapid filtration method reported previously [4,5].

Capillary-based immunoassay

The cell lysates and membrane vesicles prepared from the transfected HEK293T cells were separated and analyzed using an automated capillary electrophoresis system (Simple Western system and Compass software; ProteinSimple, Santa Clara, CA). Wes Separation Capillary Cartridges for 66–440 kDa were used for HA-BSEP and ATP1A1, respectively. The primary antibodies used were rat anti-HA (1:1000; 3F10, Roche, Mannheim, Germany) and mouse anti-ATP1A1 (1:150; C464.6, Santa Cruz Biotechnology, Dallas, TX). Signals were detected with horseradish peroxidase-conjugated secondary antibodies and visualized using ProteinSimple software.

Supplementary Figure legends

Supplementary Figure S1. Effect of meal timing on PB systemic exposure after oral administration of NaPB in Patient 1.

NaPB (150 mg/kg) was administered orally to Patient 1 30 min before, just before (<10 min), during, and just after (<10 min) breakfast following an overnight fast. Each regimen was separated by a washout period of more than 24 h. Plasma concentrations of PB were determined at the times shown. The inset depicts the same data on a logarithmic scale. Plasma concentrations of PB at 300 min after the preprandial dosing of NaPB were below the lower limit of quantification. BF, breakfast.

Supplementary Figure S2. Uncropped blot images shown in Figure 6B and C.

References

1 Hasegawa, Y. *et al.* Intractable itch relieved by 4-phenylbutyrate therapy in patients with progressive familial intrahepatic cholestasis type 1. *Orphanet J Rare Dis* **9**, 89, doi:10.1186/1750-1172-9-89 (2014).

2 Naoi, S. *et al.* Improved liver function and relieved pruritus after 4-phenylbutyrate therapy in a patient with progressive familial intrahepatic cholestasis type 2. *J Pediatr* **164**, 1219-1227 e1213, doi:10.1016/j.jpeds.2013.12.032 (2014).

3 Hayashi, H. & Sugiyama, Y. Short-chain ubiquitination is associated with the degradation rate of a cell-surface-resident bile salt export pump (BSEP/ABCB11). *Mol Pharmacol* **75**, 143-150, doi:10.1124/mol.108.049288 (2009).

4 Hayashi, H., Takada, T., Suzuki, H., Akita, H. & Sugiyama, Y. Two common PFIC2 mutations are associated with the impaired membrane trafficking of BSEP/ABCB11. *Hepatology* **41**, 916-924 (2005).

5 Hayashi, H. *et al.* Transport by vesicles of glycine- and taurine-conjugated bile salts and taurolithocholate 3-sulfate: a comparison of human BSEP with rat Bsep. *Biochim Biophys Acta* **1738**, 54-62 (2005).

Supplementary Table. Pharmacokinetic parameters of NaPB given orally at 150 mg/kg in Patient 1

Regimen	30 min before BF	Just before BF	During BF	Just after BF
C_{max} (μg/mL)	293.0	422.0	108.0	87.6
AUC_{0-4} (μg×h/mL)	373.0	511.6	178.7	109.9
T_{max} (h)	0.50	0.50	0.50	0.50
k_{el} (h^{-1})	2.70	2.40	0.66	0.60
$t_{1/2}$ (h)	0.26	0.29	1.05	1.16

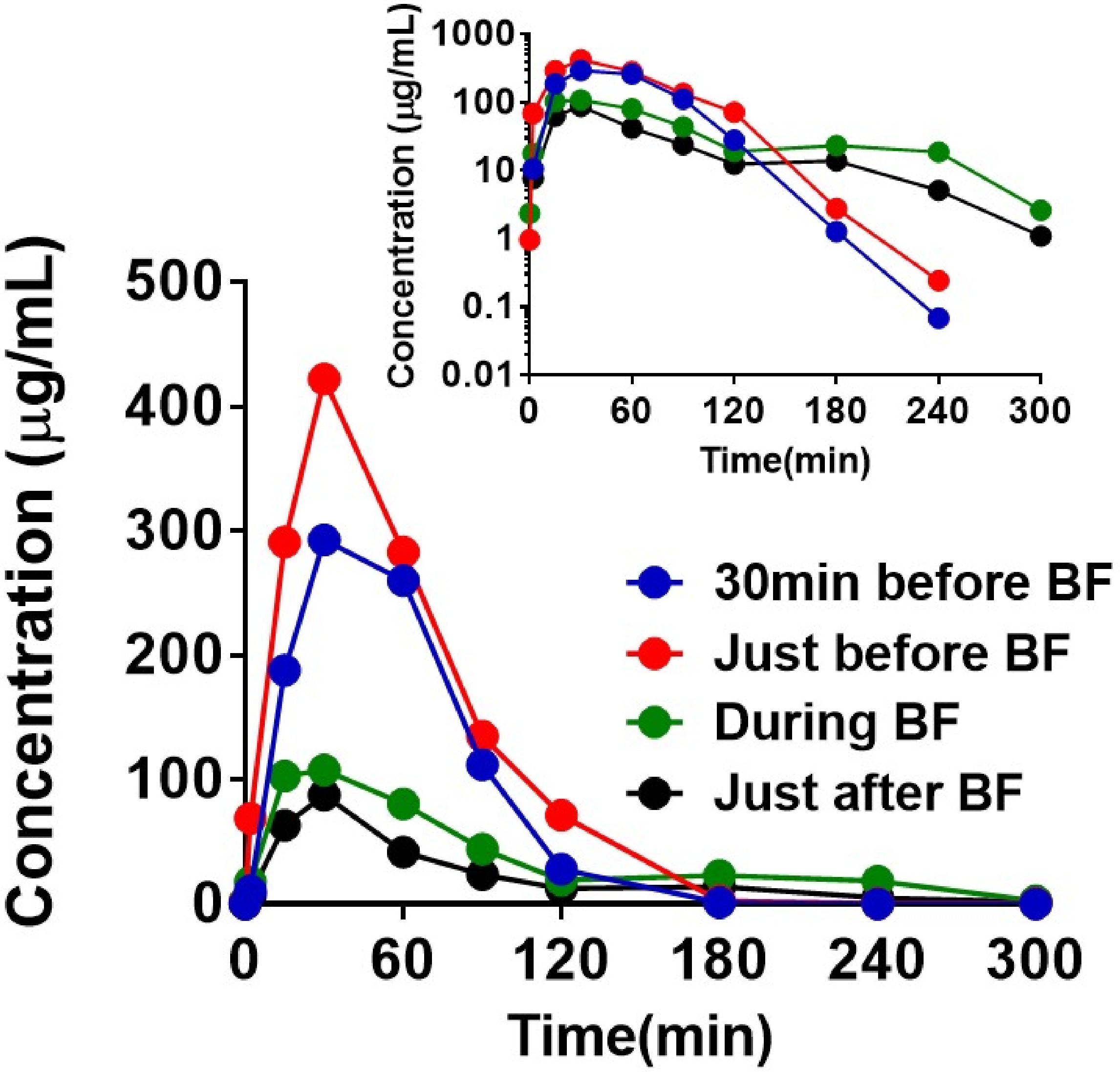

Concentration (μg/mL)
1000
100
10
1
0.1
0.01
0
60
120
180
240
300
Time(min)
Concentration (μg/mL)
500
400
300
200
100
0
0
60
120
180
240
300
Time(min)
30min before BF
Just before BF
During BF
Just after BF

Figure S1

Figure 6B

BSEP

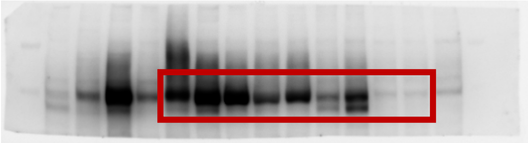

ATP1A1

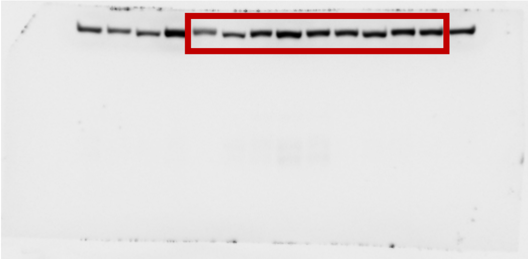

Figure 6C

BSEP

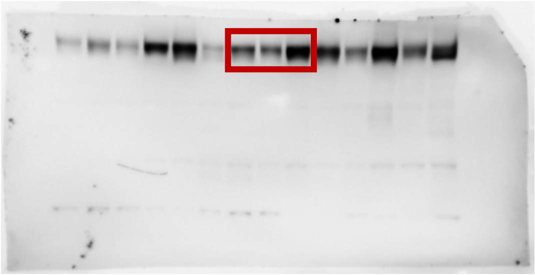

ATP1A1

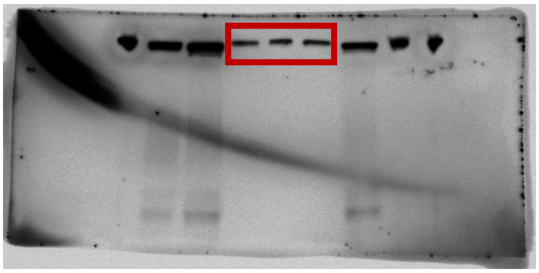

Figure S2
